# Heme oxygenase-1 inhibition mediates Gas6 to enhance bortezomib-sensitivity in multiple myeloma via ERK/STAT3 axis

**DOI:** 10.18632/aging.102996

**Published:** 2020-04-16

**Authors:** Zhaoyuan Zhang, Weili Wang, Dan Ma, Jie Xiong, Xingyi Kuang, Siyu Zhang, Qin Fang, Jishi Wang

**Affiliations:** 1 Department of Hematology, Affiliated Hospital of Guizhou Medical University, Guiyang 550004, China; 2 Key Laboratory of Hematological Disease Diagnostic and Treat Centre of Guizhou Province, Guiyang 550004, China; 3 Guizhou Province Hematopoietic Stem Cell Transplantation Centre, Guiyang 550004, China; 4 Department of Pharmacy, Affiliated Hospital of Guizhou Medical University, Guiyang 550004, China

**Keywords:** heme oxygenase-1, Gas6, bortezomib-sensitivity, ERK/STAT3 axis, multiple myeloma

## Abstract

Chemoresistance is still a critical challenge for efficient treatment of multiple myeloma (MM) during the bortezomib-based chemotherapy. Recent studies have suggested that heme oxygenase-1 (HO-1) is involved in apoptosis, proliferation and chemoresistance in cancer cells. Here we aim to investigate the role and mechanism of HO-1 in bortezomib-sensitivity to myeloma cells. In the study population, we found that HO-1 was highly expressed in CD138^+^ primary myeloma cells, which was positively associated with Gas6 expression and Gas6 plasma levels in MM patients. Downregulation of HO-1 using pharmacological inhibitor ZnPPIX or siRNA knockdown significantly enhanced myeloma cell sensitivity to bortezomib in human primary CD138^+^ cells, U266 and RPMI8226 cell lines. Mechanistically, HO-1 regulated Gas6 production via ERK/STAT3 axis. Combination with HO-1 inhibition increased bortezomib-induced apoptosis and antiproliferative effects via suppressing Gas6 production. These findings suggest that combination of bortezomib and HO-1 inhibitor may serve as a promising therapeutic target against bortezomib-resistant MM.

## INTRODUCTION

Multiple myeloma (MM) is the second most prevalent hematological malignancy, characterized by aberrant proliferation of plasma cells within the bone marrow [1]. It can cause multiple organ dysfunctions such as renal insufficiency, anemia, frequent infection, hypercalcemia and bone destruction, which has become an increasing public health burden [2]. Although the existing therapeutic drugs against MM have shown greatly remission in the large majority of patients with newly diagnosed MM, many patients still relapse due to drug resistance [3, 4]. Bortezomib, the proteasome inhibitor, is a frontline drug in the treatment of MM. The amount of MM patients who receive bortezomib-based therapeutic regimens is more than 80%, yet most of them inevitably develop acquired resistance to bortezomib, which has been a critical challenge in clinic [5]. Thus, there is an urgent need in clinic for understanding the mechanism of bortezomib resistance and identifying the effective target against this resistance.

Heme oxygenase-1 (HO-1), an enzyme catalyzing heme degradation, plays important role in regulating oxidative stress, apoptosis, proliferation, angiogenesis and chemoresistance in cancer cells [5–7]. Additionally, recent studies focused on the non-canonical function of HO-1, and showed that it may serve as an important factor to directly regulate cellular signal proteins [8–10]. Interestingly, our previous studies and others indicated that inhibition of HO-1 can decrease chemoresistance to MM [11, 12], and upregulation of HO-1 is associated with chemoresistance [13]. However, the underlying mechanisms between HO-1 and chemoresistance remain unclear.

Growth arrest-specific gene 6 (Gas6) is a 75 kDa secreted protein containing an N-terminal gamma-carboxyglutamic acid domain [14]. Gas6 has been implicated in promoting proliferation, migration and survival of multiple types of cancer cells, including breast, melanoma, ovarian, renal and prostate cancers [15–19]. Besides, emerging evidence indicate that high expression of Gas6 is associated with a poor prognosis in hematologic malignancies. Patients with acute myeloid leukemia expressing Gas6, especially those aged ≥60 years, more often fail to achieve a complete remission and have shorter disease-free and overall survive than those without Gas6 expression [20]. In addition, aberrant Gas6 secretion promotes acute myeloid leukemia cells growth and chemoresistance [21]. It suggests that Gas6 represents a critical factor for chemoresistance. However, whether HO-1 mediates the sensitivity of MM to bortezomib via Gas6 is still unknown and remains to be investigated.

We hypothesized that HO-1 inhibition downregulates Gas6 to overcome adaptive bortezomib resistance in MM, providing a potential target against drug resistance in the treatment of MM. Additionally, we aimed at investigating the molecular mechanism how HO-1 regulates Gas6 in bortezomib-resistant myeloma cells.

## RESULTS

### High HO-1 expression is associated with increased Gas6 expression in patients with MM

**Figure 1:**
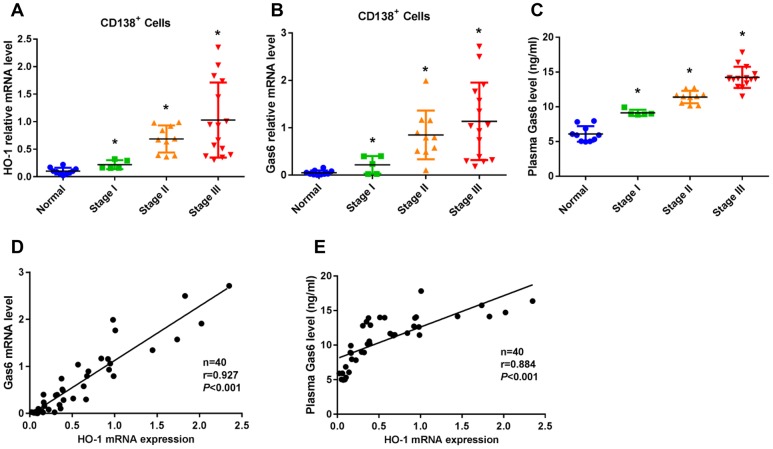
**HO-1 expression is associated with increased Gas6 expression in CD138^+^ cells from patients with multiple myeloma (MM).** (**A**, **B**) mRNA expression of HO-1 and Gas6 in human primary CD138^+^ cells were measured by qRT-PCR. β-actin was used as a control. (**C**) ELISA assay showed the plasma Gas6 level. ^*^P<0.01 vs. normal group. (**D**, **E**) A Pearman correlation analysis showed the association between HO-1 mRNA expression level and Gas 6 mRNA level (r=0.927; *P*<0.001) or Gas6 plasma level (r=0.884; *P*<0.001).

To evaluate the relationship between the expression of HO-1 and Gas6, we assessed HO-1 and Gas6 expression levels in CD138^+^ cells of bone marrow from patients at different stages of MM. Ten healthy donors and thirty patients with newly diagnosed MM, including ISS stage I (n=5), stage II (n=10) and stage III (n=15), entered into this study. The characteristics of MM patients were presented in Supplementary Table 1. HO-1 expression was found to be higher in CD138^+^ cells from MM patients than those healthy donors (*P*<0.01, Figure 1A). Similar findings were also observed in Gas6 mRNA expression (*P*<0.01, Figure 1B). Additionally, plasma levels of Gas6 were progressively elevated with increasing levels from stage I to III of MM patients (*P*<0.01, Figure 1C). Moreover, a Pearman correlation analysis showed that both mRNA level (*r*=0.927; *P*<0.001) and plasma level (*r*=0.884; *P*<0.001) of Gas6 were significantly associated with the level of HO-1 mRNA expression (Figure 1D, 1E).

### HO-1 inhibition enhances myeloma cell sensitivity to bortezomib

**Figure 2:**
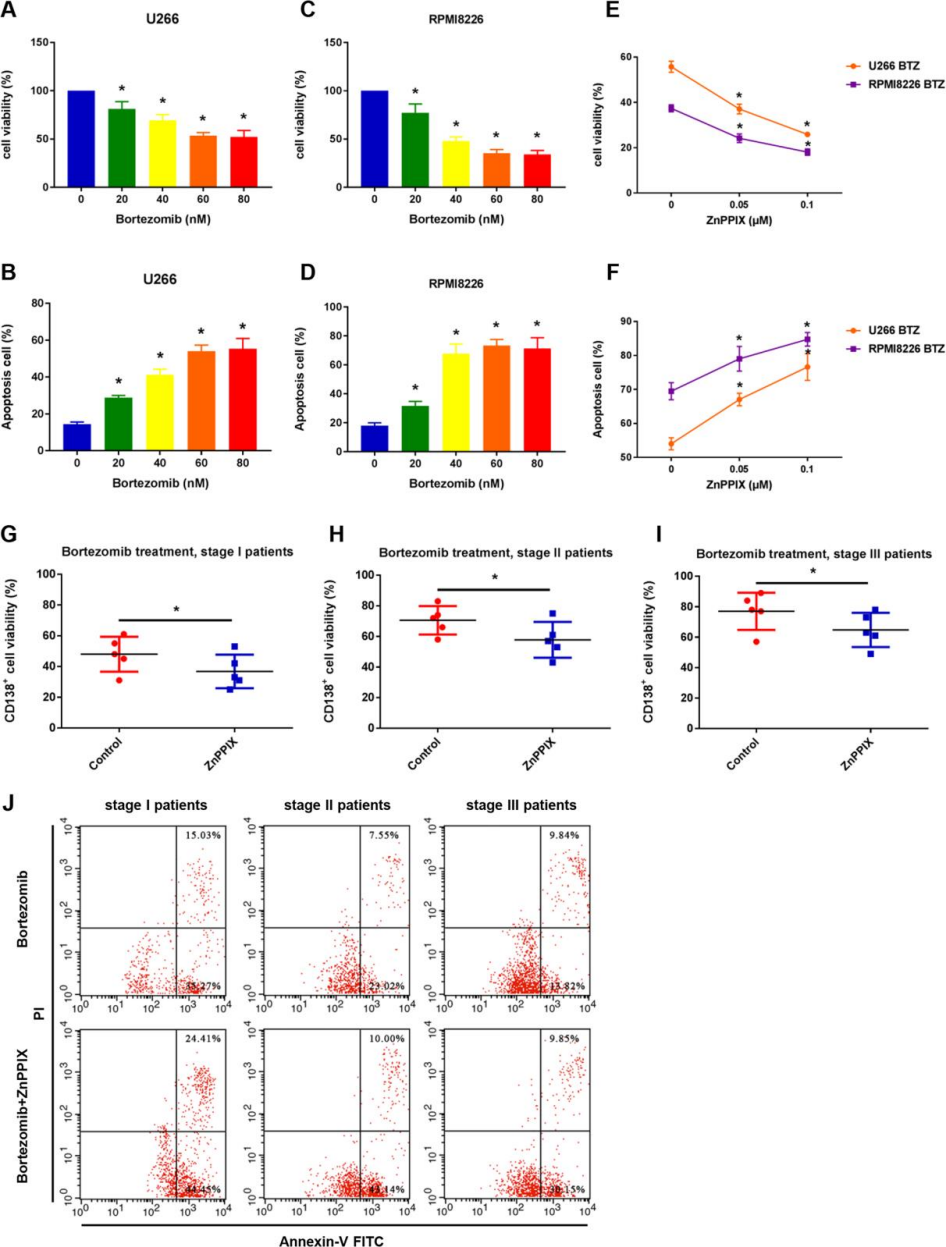
**HO-1 inhibition enhances myeloma cell sensitivity to bortezomib.** (**A**, **B**) Effects of bortezomib on the viability and apoptosis of U266 cell. Cells were treated with the indicated doses of bortezomib for 24 h and the percentage of cell viability was assessed by the CCK8 assay relative to an untreated control. (**C**, **D**) Effects of bortezomib on the viability and apoptosis of RPMI8226 cell. (**E**, **F**) Effects of ZnPPIX on the viability and apoptosis of myeloma cells in the presence of bortezomib. Cells were treated with the indicated doses of ZnPPIX plus bortezomib for 24 h. Data are presented as mean ± SD (n = 4). ^*^P < 0.05 vs. control group (0 nM). (**G**–**I**) CCK8 showing the cell viability of human primary CD138^+^ cells treated with bortezomib alone (control group) or bortezomib plus ZnPPIX. n=5, ^*^P<0.05. (**J**) Cell apoptosis (Q2+Q3) of human primary CD138^+^ cells assessed by flow cytometry after treatment with or without ZnPPIX for 24 h.

To determine the effect of bortezomib on cytotoxicity in human MM cell lines (U266 and RPMI8226), we treated cells with increasing concentrations of bortezomib for 24 hours. As shown in Figure 2A, 2B, U266 cell viability was reduced by approximately 50% and cell apoptosis rate reached approximately 50% at a dose of 60 nM. In RPMI8226 cells, the inhibition of cell viability and cell apoptosis rate were more than 50% at a dose of 40 nM (Figure 2C, 2D). Thus, the dose of 60 nM and 40 nM were chosen for U266 and RPMI8226 cells in the following bortezomib treatment, respectively. We next evaluated effect of HO-1 inhibition combined with bortezomib on MM cells using HO-1 inhibitor ZnPPIX. The results showed that cell viability was inhibited and cell apoptosis was enhanced in a dose-dependent manner upon treatment with increasing concentrations of ZnPPIX (Figure 2E, 2F). Moreover, cell viability was also significantly decreased in human primary CD138^+^ cells treated with ZnPPIX compared with the group without ZnPPIX treatment (Figure 2G–2I). In addition, ZnPPIX increased apoptosis of CD138^+^ cells from stage I, stage II and stage III MM patients by 18.56%, 22.57% and 16.34%, respectively, compared with those without ZnPPIX treatment (Figure 2J). These results suggest that HO-1 inhibitor enhances myeloma cell sensitivity to bortezomib.

### HO-1 regulates Gas6 expression via ERK/STAT3 axis

**Figure 3:**
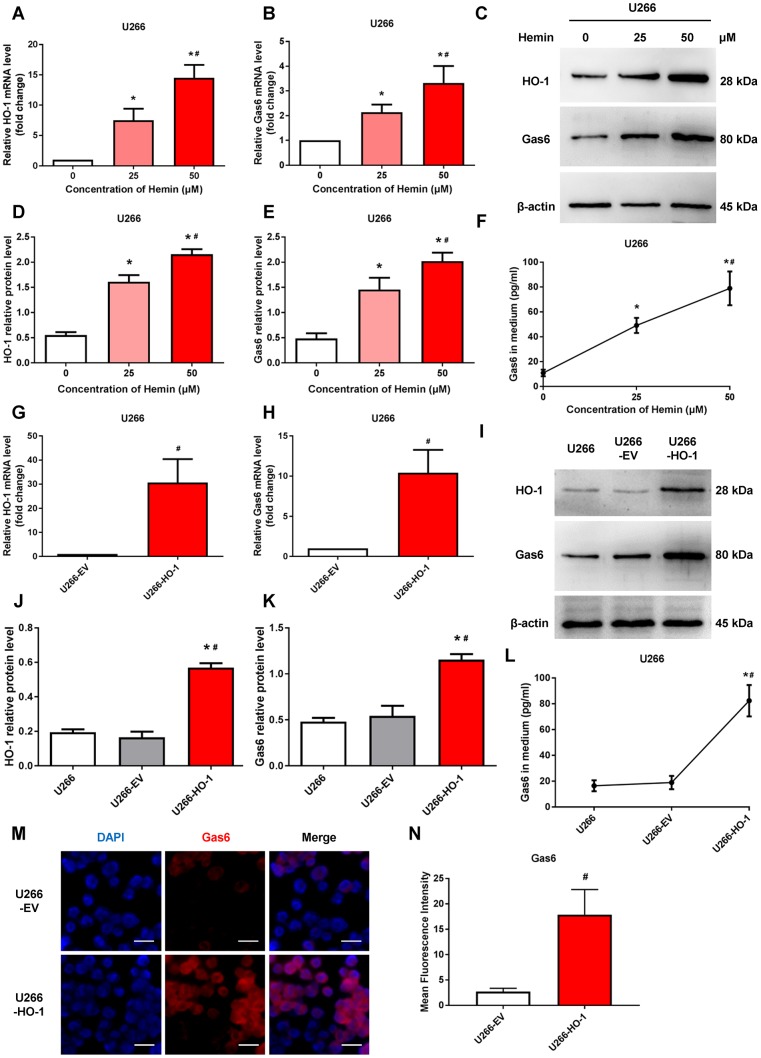
**HO-1 increases Gas6 expression in U266 cells.** (**A**, **B**) mRNA expression of HO-1 and Gas6 in U266 cells were measured by qRT-PCR. β-actin was used as a control. Cells were treated with the indicated doses of hemin for 24 h. (**C**–**E**) Western blot and semi-quantitative analysis of HO-1 and Gas6 protein levels in U266 cells-treated with hemin for 24 h. β-actin was used as a loading control. (**F**) Gas6 protein in culture supernatants from U266 cells were quantified by Gas6 ELISA. Data are expressed as mean ± SD (n = 4). ^*^P < 0.05 vs. untreated control group (0 μM); ^#^*P* < 0.05 vs. low hemin group (=25 μM). (**G**, **H**) HO-1 and Gas6 mRNA levels in U266 cells were measured by qRT-PCR after transfection with empty vector (EV) and HO-1 recombinant lentiviral. (**I**–**K**) Western blot analysis was performed to detect the protein expression of HO-1 and Gas6 in HO-1 overexpressing U266 cells. (**L**) ELISA assay showing the level of Gas6 protein in culture supernatants. (**M**, **N**) Immunofluorescence staining was performed to visualize Gas6 expression using a primary rabbit antibody against Gas6, and followed by Alexa Fluor 555-conjugated secondary antibody. The endogenous Gas6 was shown in red. Nuclei were stained with DAPI (blue). The scale bars represent 100 μm. Data are expressed as mean ± SD (n = 4). ^*^P < 0.05 vs. U266 group; ^#^*P* < 0.05 vs. U266-EV group.

**Figure 4:**
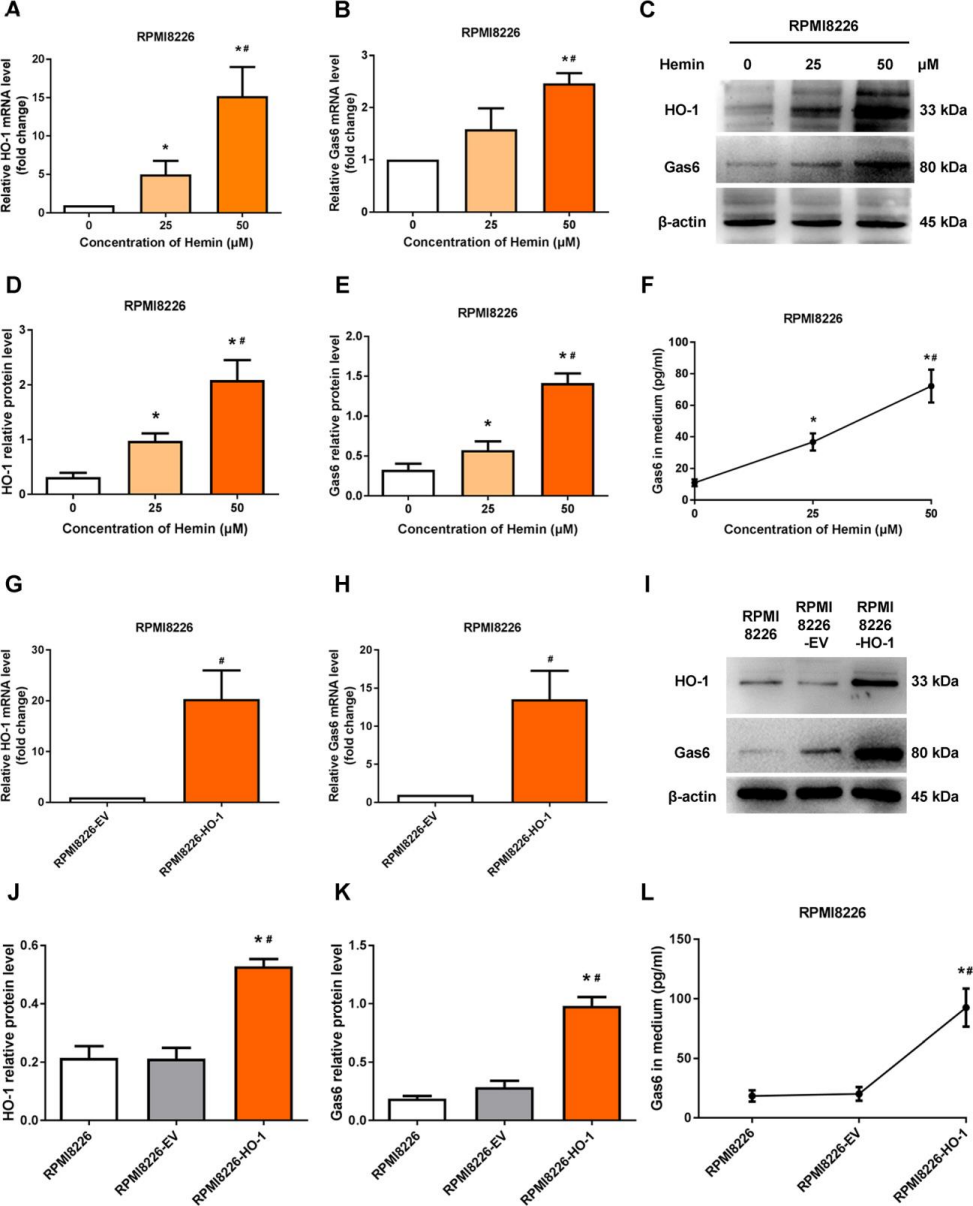
**HO-1 upregulates Gas6 expression in RPMI8226 cells.** (**A**, **B**) mRNA expression of HO-1 and Gas6 in RPMI8226 cells were measured by qRT-PCR. (**C**-**E**) Western blot and semi-quantitative analysis of HO-1 and Gas6 protein levels in RPMI8226 cells-treated with hemin for 24 h. (**F**) Gas6 protein in culture supernatants from RPMI8226 cells were measured by Gas6 ELISA. (**G**, **H**) HO-1 and Gas6 mRNA levels in RPMI8226 cells were measured by qRT-PCR after transfection with empty vector (EV) and HO-1 recombinant lentiviral. (**I**–**K**) The effects of HO-1 overexpression on Gas6 protein expression level was shown in RPMI8226 cells. (**L**) The effects of HO-1 overexpression on Gas6 secretion in culture supernatants from RPMI8226 cells. Data are expressed as mean ± SD (n = 4). ^*^P < 0.05 vs. RPMI8226 group; ^#^*P* < 0.05 vs. RPMI8226-EV group.

We next determine whether HO-1 regulates Gas6 expression in MM cells. U266 cells were treated with hemin to induce the expression of HO-1. Western blotting and qRT-PCR showed the upregulation of HO-1 with different concentration of hemin treatment (Figure 3A–3D). Then, we exposed U266 cells to increasing concentrations of hemin for 24 hours. As shown in Figure 3B–3E, compared with the control group cultured without hemin, the mRNA and protein levels of Gas6 were increased in U266 cells treated with increasing hemin concentrations (from 25 to 50 μM; *P*<0.01). Simultaneously, the secretion of Gas6 was gradually elevated with increasing concentrations of hemin in U266 cells (*P*<0.01; Figure 3F). Furthermore, HO-1-overexpressing myeloma cell line U266-HO-1 and its corresponding controls were established. Western blotting and qRT-PCR were used to verify transfection efficiency (Figure 3G, 3I–3J). Not only the mRNA and protein levels but also the secretion of Gas6 was significantly elevated by HO-1 overexpression (Figure 3H, 3K, 3L). Additionally, immunofluorescence analysis showed that Gas6 expression was marked increase in U266-HO-1 cells compared with U266-EV cells (Figure 3M, 3N). Similar results were also found in RPMI8226 cells (Figure 4). Moreover, we inhibited HO-1 by using a pharmacological inhibitor ZnPPIX, and knock down HO-1 gene by siRNA. The findings that HO-1 inhibition suppressed Gas6 expression and secretion were observed in myeloma cell (Supplementary Figures 1, 2).

**Figure 5:**
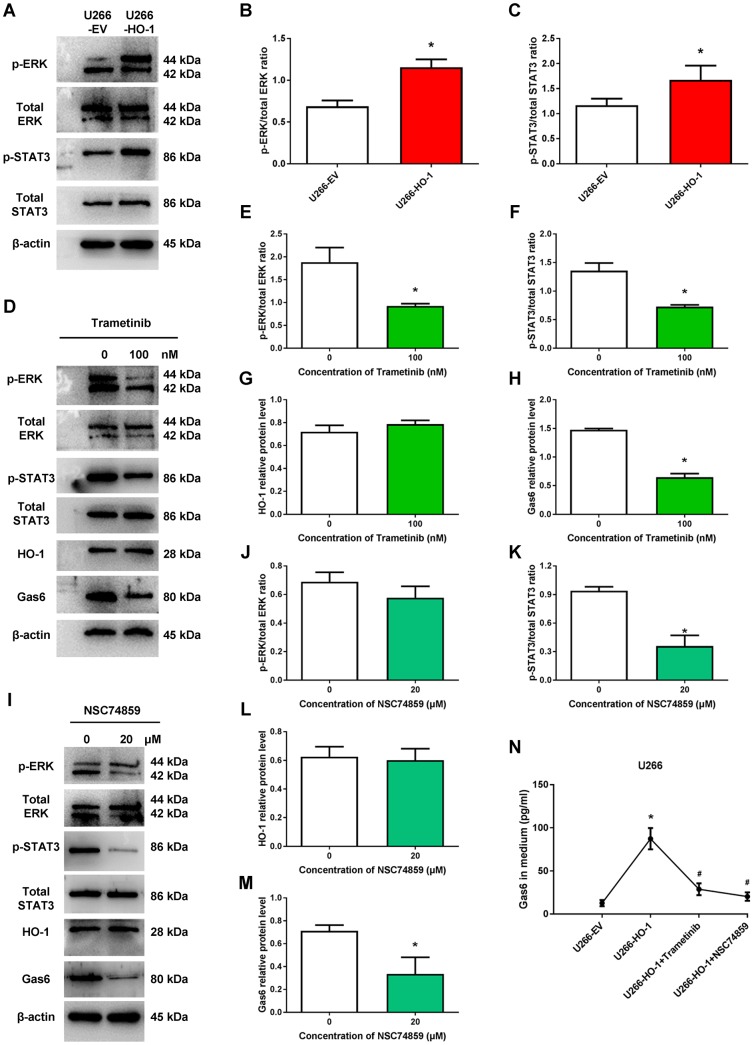
**ERK/STAT3 axis is involved in HO-1-mediated Gas6 expression.** (**A**–**C**) The effects of HO-1 overexpression on the phosphorylation level of ERK and STAT3 were determined by Western blot. n=4, ^*^P<0.05 vs. U266-EV. (**D**–**H**) The effects of ERK inhibitor trametinib on the phosphorylation level of ERK and STAT3, and the protein expression of HO-1 and Gas6. n=4, ^*^P<0.05 vs. the group treated without trametinib (=0 nM). (**I**–**M**) The effects of STAT3 inhibitor NSC74859 on the phosphorylation level of ERK and STAT3, and the protein expression of HO-1 and Gas6. n=4, ^*^P<0.05 vs. the group treated without NSC74859. (**N**) Gas6 levels in culture supernatants from U266 cells were quantified by Gas6 ELISA. Cells were treated with empty vector (EV), HO-1 recombinant lentiviral, trametinib or NSC74859. n=4, ^*^P<0.05 vs. U266-EV; ^#^*P*<0.05 vs. U266-HO-1.

Previous study demonstrated the importance of STAT3 in promoting chemoresistance of cancer cells via transcriptional regulation [22]. Recent evidence revealed that Gas6 effect was STAT3-dependent [23]. Thus, to determine the mechanism by which HO-1 enhanced the expression of Gas6 in MM cells, we evaluated the expression of STAT3 and the related signal pathway. The results showed that the phosphorylation level of ERK and STAT3 were increased by HO-1 overexpression, respectively (Figure 5A–5C). Interestingly, we found that ERK inhibitor trametinib significantly reduced the expression of Gas6 and the ratio of p-STAT3/total STAT3, but did not influence HO-1 expression (Figure 5D–5H). However, the effect that Gas6 enhanced by HO-1 was block by STAT3 inhibitor NSC74859, whereas it had no significant effect on the expression of HO-1 and the ratio of p-ERK/total ERK (Figure 5I–5M). Beside, we observed that both trametinib and NSC74859 markedly decreased the production of soluble Gas6 in culture medium from myeloma cells (Figure 5N). These data reinforce our hypothesis that HO-1 regulates Gas6 production via ERK/STAT3 axis.

### HO-1 inhibition enhances bortezomib-induced anti-proliferative effect by reducing Gas6 secretion

**Figure 6:**
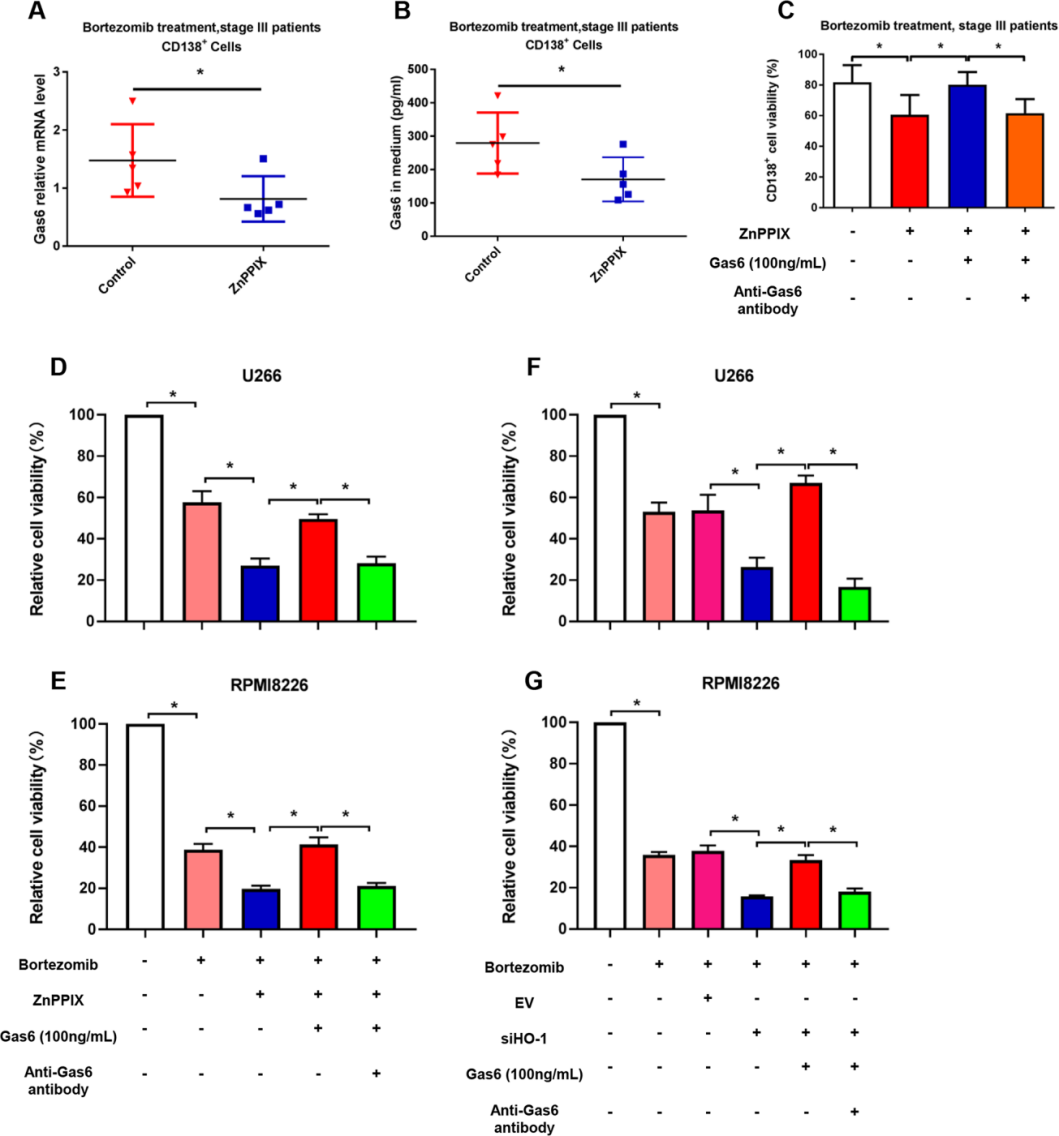
**HO-1 inhibition enhances bortezomib-induced antiproliferative effect by reducing Gas6 secretion.** (**A**) Gas6 mRNA level in human primary CD138^+^ cells. (**B**) Gas6 levels of culture supernatants were quantified by ELISA. Human primary CD138^+^ cells were treated with or without ZnPPIX (0.1 μM) in the presence of bortezomib for 24 h. n=5, ^*^P<0.05 vs. control group (ZnPPIX = 0 μM). (**C**) CCK8 showing the cell viability of CD138^+^ cells treated with ZnPPIX, exogenous Gas6 or Gas6 neutralizing antibody. n=5, ^*^P<0.05. (**D**–**G**) Cell viability was detected by CCK8 assays in U266 and RPMI8226 cells. n=4, ^*^P<0.05.

Our findings showed that HO-1 inhibitor ZnPPIX enhances myeloma cell sensitivity to bortezomib (Figure 2). Interestingly, such processes were accompanied with a decrease in Gas6 mRNA level and the secretion of Gas6 in culture medium after ZnPPIX treatment (Figure 6A, 6B). To investigate whether the effect that HO-1 inhibition enhances bortezomib-sensitivity is mediated by Gas6 secretion, human primary CD138^+^ plasma cells were exposed to exogenous Gas6 for 24 hours. The CCK8 results showed that exogenous Gas6 significantly increased CD138^+^ cells viability, and this effect was reversed by Gas6 neutralizing antibody (Figure 6C). In addition, we also use ZnPPIX or HO-1 siRNA to inhibit HO-1 expression in U266 and RPMI8226 cells. As shown in Figure 6D–6G, exogenous Gas6 significantly blocked anti-proliferative effect enhanced by ZnPPIX or HO-1 siRNA, and Gas6 neutralizing antibody abolished pro-proliferation induced by Gas6.

### Combination with HO-1 inhibition enhances bortezomib-induced apoptosis via suppressing Gas6 production

**Figure 7:**
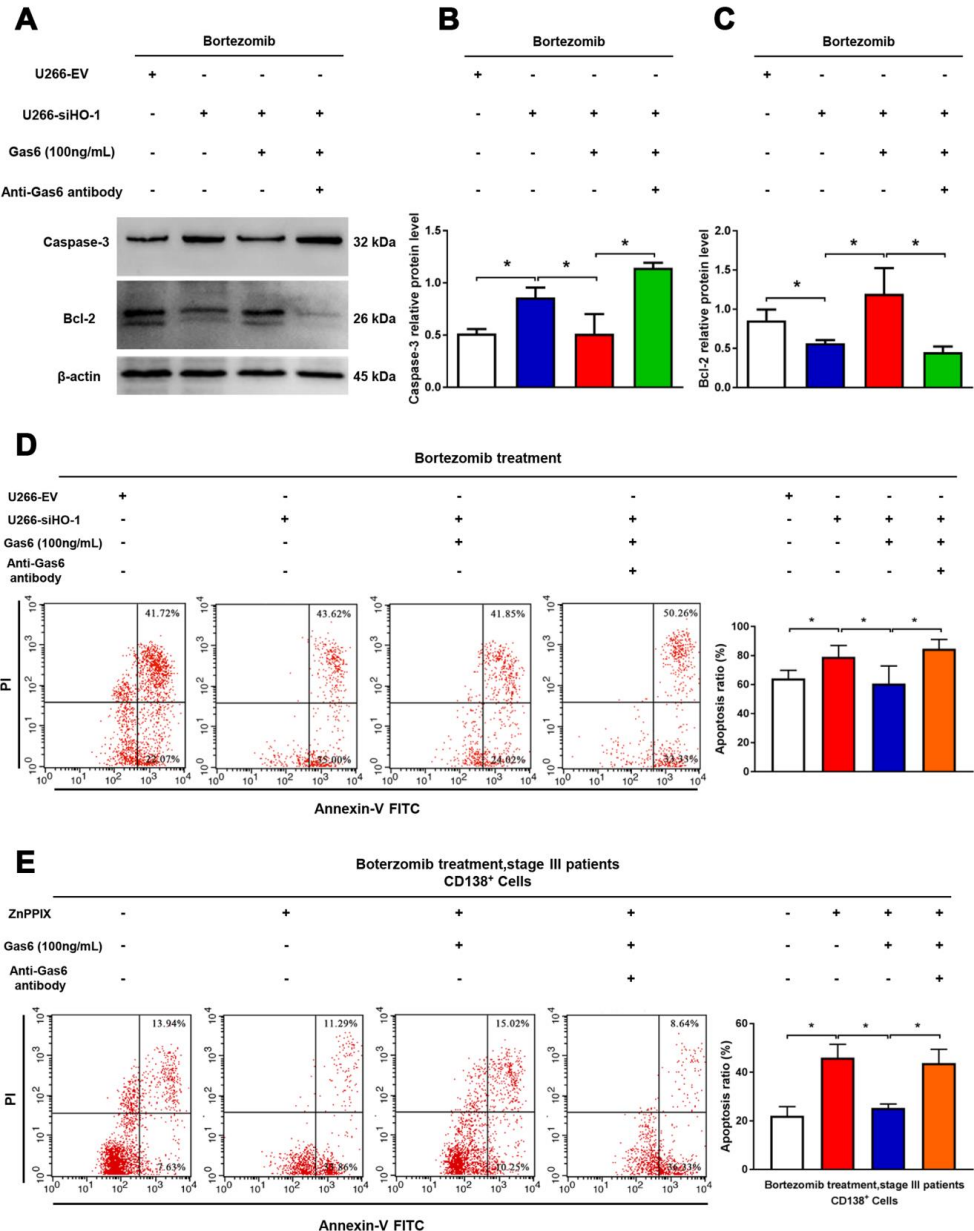
**Combination with HO-1 inhibition enhances bortezomib-induced apoptosis via suppressing Gas6 production.** (**A**–**C**) Caspase-3 and Bcl-2 relative protein levels were detected by western blot analysis. n=3, ^*^P<0.05. (**D**) Cell apoptosis (Q2+Q3) of U266 cells assessed by flow cytometry after transfection with HO-1 siRNA and treatment with or without exogenous Gas6 or Gas6 neutralizing antibody for 24 h. n=4, ^*^P<0.05. (**E**) Flow cytometry was used to assess human CD138^+^ cells apoptosis after treatment with HO-1 inhibitor ZnPPIX with or without exogenous Gas6 or Gas6 neutralizing antibody for 24 h. n=3, ^*^P<0.05.

As shown in Figure 7, combination bortezomib and HO-1 inhibition treatment significantly aggravated U266 cell apoptosis compared with bortezomib alone. Exogenous Gas6 inhibited pro-apoptosis effect enhanced by HO-1 inhibition with a decrease in pro-apoptotic protein caspase-3 expression but an increase in anti-apoptotic protein Bcl-2 expression, and Gas6 neutralizing antibody reversed anti-apoptosis induced by Gas6 (Figure 7A–7D). Similar findings were also observed in human primary CD138^+^ cells (Figure 7E). Collectively, these results suggest that HO-1 inhibition enhance sensitivity to bortezomib in myeloma cells via suppressing Gas6 production.

## DISCUSSION

In this study, our main findings demonstrate that HO-1 is associated with increased Gas6 expression in MM patients, and inhibition of HO-1 enhances sensitivity to bortezomib in myeloma cells via suppressing Gas6 expression and secretion.

Although bortezomib is usually effective in patients with newly diagnosed MM, MM remains an incurable disease due to inevitable drug resistance [24, 25]. Therefore, it is essential in our efforts to find a potential target against bortezomib resistance. In the present study, we have isolated and compared the expression of HO-1 in CD138^+^ myeloma cells from healthy donors and patients with different stages of MM. We have demonstrated that HO-1 expression levels were progressively elevated from stage I to III of MM patients. HO-1 was previously found to mediate tumor microenvironment for cancer cell growth, angiogenesis, metastasis and chemoresistance [6, 26]. Our previous studies showed upregulation of HO-1 expression in chemotherapy-resistant myeloma cells and marrow stromal cells [12, 27]. Giovanni LV et al. reported that HO-1 was upregulated in various MM cell lines, and they hypothesized that the HO-1 protein itself, rather than its enzymatic activity, could play a major role in some hematological malignancies [28]. Our current study was consistent with these observations. More importantly, our current study advanced significantly from previous studies. We further showed that inhibition of HO-1 by siRNA knockdown or pharmacological approach with ZnPPIX could enhance sensitivity to bortezomib in MM cell lines and human primary CD138^+^ myeloma cells. Nonetheless, the underlying mechanisms by which HO-1 involves in the process of bortezomib-resistance need to be further investigated.

Immune escape of tumor plasma cells plays an important role in MM progression and drug-resistant phenotype [29]. Recent evidence shows that bone marrow microenvironment promotes MM cells to escape the immune system [30]. Specifically, under hypoxic and stress conditions, tumor plasma cells inhibit anti-tumor immune effectors, including T cells, NK cells and antigen-presenting dendritic cells, while simultaneously promoting the immunosuppressive and pro-tumor properties of myeloid cells. Interestingly, this hypoxic microenvironment may also upregulate HO-1 expression and thus further promote cell survival and drug-resistant. Gozzelino R et al. demonstrated that cellular stress resulting in the generation of ROS triggered signal transduction affecting the interaction of Bach-1 repressor/NF-E2-related transcription factor-2 with the HMOX1 promoter increasing HO-1 expression [31]. Our data showed that HO-1 upregulation was associated with resistance of MM cells to chemotherapy, which suggests that HO-1 represents a targetable novel tumor escape mechanism in MM.

Recently, Gas6 has been reported to be a crucial molecule implicated in tumor cell proliferation, anti-apoptosis, invasion and resistance to anticancer drugs [32–34]. Using heat map analysis, Furukawa M et al. showed that Gas6 was significantly upregulated in MM compared with other hematological malignancies [35]. Our results are accordance with previous studies. We observed that Gas6 was greatly increased in CD138^+^ myeloma cells from stage I to stage III MM patients compared with healthy controls. Interestingly, we found that Gas6 expression level was positively associated with HO-1 expression level in MM patients, suggesting that there may be a potential linker between HO-1 and Gas6. Until now, no evidence exists about the relationship between HO-1 and Gas6 in the development of chemoresistance in MM. In the present study, we revealed the following findings: 1) in human MM cell lines, including U266 and RPMI8226, HO-1 contributed to upregulate Gas6 expression and secretion; 2) inhibition of HO-1 downregulated Gas6 expression and secretion, and subsequently overcame bortezomib resistance in MM cells; 3) the addition of exogenous Gas6 abolished the effects enhanced by HO-1, and this phenotype could be reversed by Gas6 neutralizing antibody. Our data suggesting that Gas6 may act as an important downstream factor involved in the process of HO-1 enhanced sensitivity to bortezomib in MM.

Our data are in line with Waizenegger JS et al. presented that the level of Gas6 as secreted by MM cell lines was much lower than plasma Gas6 level of MM patients [36]. It’s possible that other type of cells within bone marrow contribute to Gas6 secretion besides MM cells. Another study found that Gas6 secretion by leukemia was low and Gas6 was mainly produced by bone marrow stroma cells [21]. Our results showed that HO-1 enhanced autocrine action of Gas6 in MM cell lines and CD138^+^ myeloma cells, but the function of HO-1 in Gas6 secretion by bone marrow stroma cells is unclear. Further studies need to explore whether HO-1 regulate bone marrow stroma cells to produce Gas6 and unravel the functions of HO-1 in MM cells versus stroma cells-derived Gas6 in MM.

**Figure 8:**
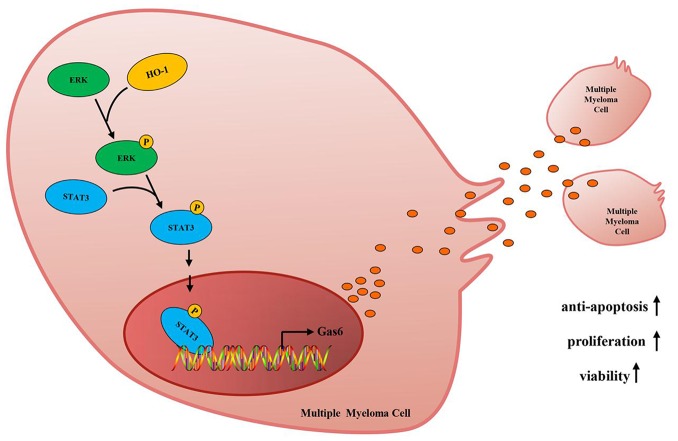
**Mechanism diagram. HO-1 up-regulates Gas6 to mediate the sensitivity of myeloma cells to bortezomib via activating ERK/STAT3 signaling pathway.**

ERK1/2 signaling is involved in regulating a variety of cancer cells functions, such as cell proliferation, migration, differentiation and apoptosis [37–40]. Mandal R et al illustrated that ERK1/2 signaling was activated in about half of MM patients and was considered to be a potential target in MM [41]. Another study demonstrated that activated ERK1/2 phosphorylated downstream substrates, such as transcription factors, that controlled the development of cancer [42]. Recently, it has been suggested that Gas6 expression is mediated by transcription factor STAT3 in pro-inflammatory condition [23]. However, whether ERK/STAT3 axis is involved in the process of HO-1 enhanced Gas6 expression in MM has not been explored. In present study, we found that HO-1 activated ERK/STAT3 signaling in MM cells. Using the ERK1/2 signaling inhibitor trametinib significantly reduced HO-1 enhanced Gas6 and STAT3 expression, but not in HO-1 expression. Furthermore, we observed that STAT3 inhibitor NSC74859 greatly decreased Gas6 expression, whereas HO-1 and ERK1/2 phosphorylation were not affected by NSC74859. Hence, our findings suggested that HO-1 enhanced Gas6 expression and subsequently bortezomib resistance by activating ERK/STAT3 axis (Figure 8).

In this study, we used International Staging System (ISS) to divide MM patients into different stages. Although ISS has been widely accepted as the standard prognostic model for MM patients since 2005 [43], recent evidence shows that the revised ISS (R-ISS), which incorporates cytogenetic abnormality into ISS, performs well and has better discriminative power than the ISS in MM patients [44]. This limitation in this study should be highlighted. Future studies are needed to assess the relationship between HO-1 and bortezomib-sensitivity in MM patients according to R-ISS.

In summary, our study showed that high HO-1 expression was associated with increased Gas6 level in the development of MM. HO-1 inhibition suppressed Gas6 expression and subsequent bortezomib resistance by inhibiting ERK/STAT3 axis. We further demonstrated that the combination of bortezomib and HO-1 inhibitor downregulated Gas6 to enhance sensitivity to bortezomib in MM. Our work delineates the role of HO-1/Gas6 axis in bortezomib resistance, and validates HO-1 as a promising therapeutic target in MM.

## MATERIALS AND METHODS

### Patients and samples collection

This study conformed to the ethical guidelines of the 1975 Declaration of Helsinki as reflected in a priori approval by the Ethics Committee of Guizhou Medical University. Written informed consents were obtained from each participant involved in this study before using these clinical samples for research purposes. Bone marrow tissues and peripheral blood samples were obtained from 30 newly diagnosed MM patients. Samples from 10 healthy donors were used as controls. CD138^+^ cells were purified using CD138 microbeads with Miltenyi magnetic cell sorting system (Miltenyi Biotec, CA, USA) as described previously [45]. According to the ISS, the MM patients were divided into stage I, II and III. The characteristics of MM patients were shown in Supplementary Table 1.

### Cell culture

The human MM cell lines U266 and RPMI8226 were obtained from Cobioer Bioscience (Nanjing, China) and maintained in RPMI-1640 medium (Gibco, Invitrogen, Carlsbad, CA) supplemented with 10% fetal bovine serum (Gibco, Invitrogen, Carlsbad, CA), 100 U/ml penicillin and 100 mg/ml streptomycin at 37 °C in a humidified atmosphere containing 5% CO_2_. Experiments were performed using logarithmically growing cells.

### Reagents

The HO-1 enhancer hemin was obtained from Sigma (St Louis, MO, USA). HO-1 inhibitor ZnPPIX was purchased from Cayman Chemical (Ann Arbor, MI, USA). Bortezomib, Trametinib (an ERK inhibitor) and NSC74859 (a STAT3 inhibitor) were obtained from Selleck Chemicals (Houston, TX, USA). Recombinant human Gas6 (R&D Systems), anti-Gas6 antibody (Bioworld Technology), Gas6 primary antibody (Solarbio Life Science) and mouse IgG isotype control antibody (SouthernBiotech) were used. The following primary antibodies were purchased from Cell Signaling Technology: HO-1, β-actin, total extracellular signal-regulated kinase (ERK), p-ERK, total signal transducer and activator of transcription 3 (STAT3), p-STAT3, caspase-3 and Bcl-2.

### Construction of recombinant lentiviral vectors and transfection

For the stable breakdown or overexpression of HO-1, we constructed HO-1 targeting small interfering RNA (siRNA) and HO-1 overexpression myeloma cell lines as described previously [12]. Retroviruses were generated by transfecting empty vectors containing the enhanced green fluorescence protein (EGFP) or vectors containing human HO-1-EGFP/siRNA-HO-1-EGFP into 293FT packaging cells. Four recombinant lentiviral vectors were constructed: lentivirus-V5-D-TOPO-HO-1-EGFP (L-HO-1), lentivirus-V5-D-TOPO-EGFP (TOPO-EGFP), lentivirus-pRNAi-u6.2-EGFP-siHO-1 (L-siHO-1), and lentivirus-pRnai-u6.2-EGFP (RNAi-EGFP). MM cell lines U266 and RPMI8226 infected with lentiviral vectors containing L-siHO-1 were referred to as U266-siHO-1 and RPMI8226-siHO-1; the HO-1-overexpressing myeloma cell lines infected with L-HO-1 were named U266-HO-1 and RPMI8226-HO-1; and their corresponding controls were named U266-EV and RPMI8226-EV. The infection efficacy was determined by western blot.

### RNA extraction and semi-quantitative real time-polymerase chain reaction (qRT-PCR)

Total RNA was extracted from cells with Trizol (Invitrogen, Carlsbad, CA, USA), and reverse-transcribed using the PrimeScript RT reagent kit (TaKaRa, Japan) following the manufacturer’s instructions. Amplification reaction was set up in 20 μl reaction volumes containing cDNA samples, primers and SYBR Premix Ex Taq TM II (TaKaRa, Japan). The following primers were synthesized by Transheep Biotechnology Co. LTD: HO-1, forward 5’-ACCCATGACACCAAGGACCAGA-3’ and reverse 5’-GTGTAAGGACCCATCGGAGAAGC-3’; Gas6, forward 5’-CGGATGTGAGCCACGACTT-3’ and reverse 5’-CCAGGAAACGGTGAAAGTG-3’; β-actin, forward 5’-GAGACCTTCAACACCCCAGC-3’ and reverse 5’-ATGTCACGCACGATTTCCC-3’. Each sample was run in triplicate and each experiment was repeated at least three times. Results were analyzed using the PRISM 7500 real-time PCR detection system (ABI, USA). The fold change was determined using ΔΔCt method. Gene expression was normalized using β-actin RNA.

### Western blot analysis

Cells were collected and extracted with RIPA lysis buffer (50 mM Tris HCl, 150 mM NaCl, 5 mM EDTA, 1% NP-40, 1% sodium deoxycholate, 0.1% SDS, 1% aprotinin, 50 mM NaF, 0.1 mM Na_3_VO_4_) to detect changes in cellular protein levels. Samples with equal amounts of protein were separated by SDS-PAGE electrophoresis. Separated proteins were transferred to polyvinylidene fluoride (PVDF) membranes (Millipore Corporation, MA, USA) and then incubated with the corresponding primary antibodies for HO-1 (1:1000), Gas6 (1:1000), p-ERK (1:1000), total ERK (1:1000), p-STAT3 (1:1000), total STAT3 (1:1000), Caspase-3 (1:1000), Bcl-2 (1:1000) or β-actin (1:1000) overnight at 4 °C after blocking with 5% non-fat milk. The membranes were washed and then incubated with HRP-conjugated secondary antibody (1:2000) for 2 hours at room temperature. The signal was determined by enhanced chemiluminescence (7 Sea Biotech, Shanghai, China). The expression levels of related proteins were semi-quantified and normalized against β-actin using ImageJ software (National Institutes of Health, USA).

### ELISA assay for Gas6

The levels of Gas6 were determined in cell culture supernatants and human plasma using a commercially available Gas6 ELISA kit (R&D Systems) according to the manufacturer’s instructions. Briefly, sample (100 μL) was added to a 96-well microplate coated with anti-Gas6 antibody and incubated 2 hours at room temperature. After washing, add 100 μL of Biotinylated anti-Gas6 detection antibody to each well followed by the incubation with Streptavidin-HRP for 20 minutes at room temperature. After washing, add 100 μL of substrate solution to each well and incubate for 20 minutes at room temperature. When the stop solution was added to each well, the absorbance at 450 nm was read immediately using a microplate reader (Molecular Devices, Sunnyvale, CA, USA).

### Cell viability assay

MM cell viability was assessed by the cell counting kit 8 (CCK8, Dojindo, Kumamoto, Japan) assay following the manufacturer’s instructions. Briefly, a total of 5 × 10^3^/well cells were seeded in 96-well plates with related treatment and incubated at 37 °C in a humidified atmosphere with 5% CO_2_. Each sample was determined in triplicate. 10 μL of CCK8 solution was added to each well. After 1 hour of incubation at 37 °C, the absorbance at 450 nm was measured using a spectrophotometer (Molecular Devices, Sunnyvale, CA, USA), and the cell viability was subsequently calculated.

### Immunofluorescence

U266 cells were fixed with 4% paraformaldheyde and permeabilized in 0.1% Triton X-100. After washing with PBS, cells were blocked with 5% bovine serum albumin (Sigma, USA) for 2 hours at room temperature. Thereafter, cells were incubated with primary antibody for Gas6 (1:100) overnight at 4 °C followed by the incubation with Alexa Fluor 555-conjugated secondary antibody (1:500) for 1 hour at room temperature. Cells were washed with PBS, and the nuclei stained with DAPI (Sigma, USA). Cells were observed and the images were captured using same exposure times and identical camera setting by immunofluorescence microscopy (Leica DM4000B, Wetzlar, Germany).

### Flow cytometry for apoptosis detection

MM cells apoptosis were determined using an Annexin V-FITC/PI apoptosis kit (BD Biosciences, San Jose, CA, USA) according to the manufacturer’s instructions. Human U266 cells and CD138^+^ cells with different treatments for 24 hours were harvested, washed in PBS, and resuspended in 100 μL of binding buffer. Then, 5 μL of annexin V-FITC was added into the cell suspension and incubated in the dark for 5 min followed by the incubation with PI (5 μL) for 15 min. The cells were subjected to flow cytometry, and data were analyzed by FACSCalibur (BD Biosciences).

### Statistical analysis

Data were presented as the mean ± standard deviation. Comparisons between two groups were performed with Student’s t test or non-parametric Mann-Whitney test when appropriate. One-way analysis of variance (ANOVA) was used to estimate differences between three or more groups. A Pearman correlation analysis was used to assess the strength of association between HO-1 mRNA level and Gas6 mRNA or plasma Gas6 level in patients with MM. *P* values less than 0.05 were considered statistically significant. All statistical analyses were performed using GraphPad Prism 7.0 (GraphPad Software, CA, USA).

## Supplementary Material

Supplementary Figures

Supplementary Table 1
