# Supplementary material for: Heme oxygenase-1 inhibition mediates Gas6 to enhance bortezomib-sensitivity in multiple myeloma via ERK/STAT3 axis
Source: Aging (Albany NY). 2020 Apr 16;12(8):6611–29. doi: 10.18632/aging.102996 (PMC7202511; doi:10.18632/aging.102996)
Supplement: Supplementary Figures [file aging-12-102996-s002..pdf]

SUPPLEMENTARY FIGURES

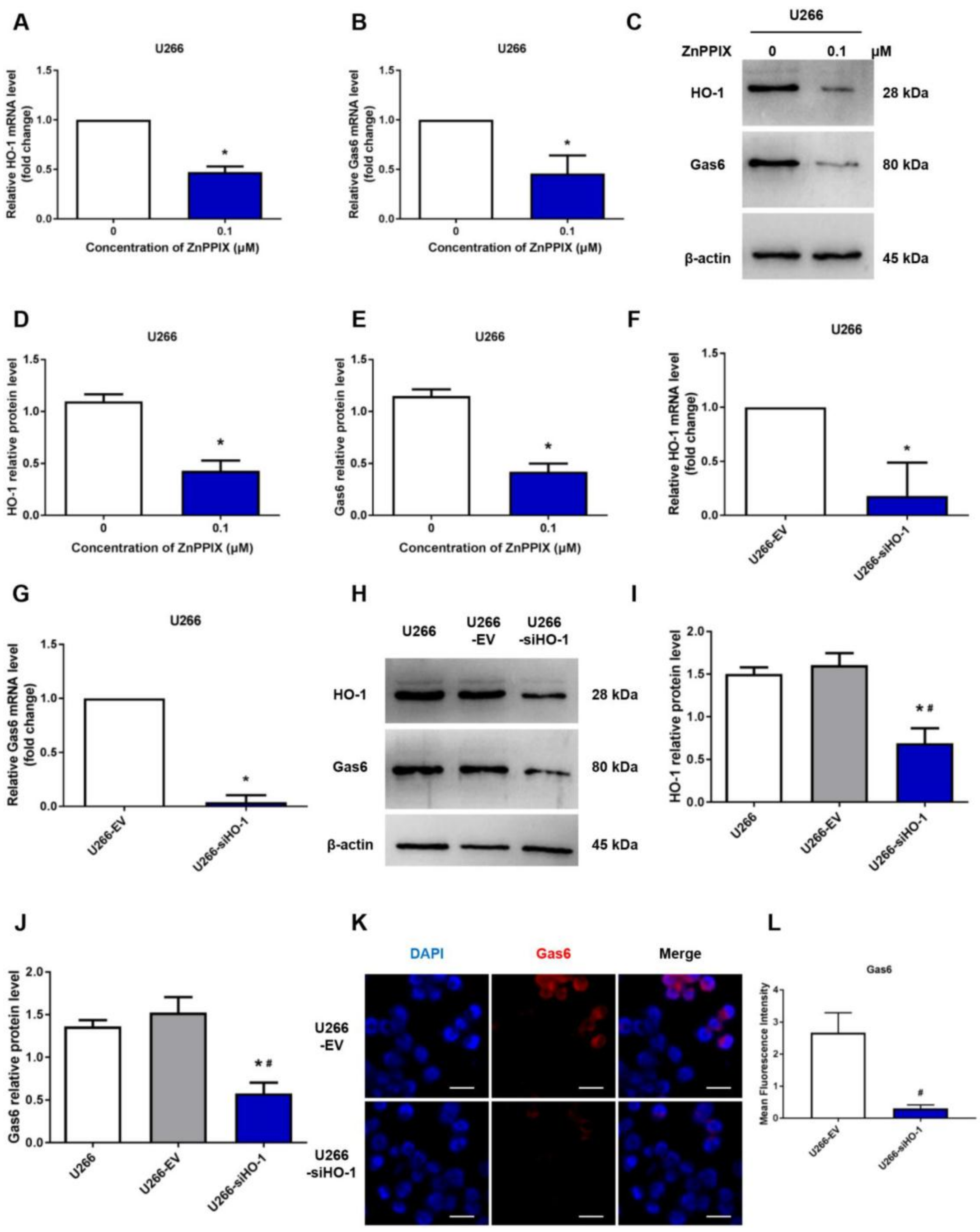

Supplementary Figure 1. HO-1 inhibition decreases Gas6 production inU266 cells. (**A**, **B**) mRNA expression of HO-1 and Gas6 in U266 cells were measured by qRT-PCR. β-actin was used as a control. Cells were treated with ZnPPIX for 24 h. (**C**–**E**) Western blot and semi-quantitative analysis of HO-1 and Gas6 protein levels in U266 cells-treated with ZnPPIX for 24 h. β-actin was used as a loading control. Data are expressed as mean ± SD (n = 4). **P*< 0.05 vs. untreated control group (0 μM). (**F**, **G**) HO-1 and Gas6 mRNA levels in U266 cells were measured by qRT-PCR after transfection with empty vector (EV) and HO-1 siRNA. (**H**–**J**) Western blot analysis was performed to detect the protein expression of HO-1 and Gas6 in HO-1 knockdownU266 cells. (**K**, **L**) Immunofluorescence staining was performed to visualize Gas6 expression using a primary rabbit antibody against Gas6, and followed by Alexa Fluor 555-conjugated secondary antibody. The endogenous Gas6 was shown in red. Nuclei were stained with DAPI (blue). The scale bars represent 100 μm. Data are expressed as mean ± SD (n = 4). **P*< 0.05 vs. U266; #*P*< 0.05 vs. U266-EV.

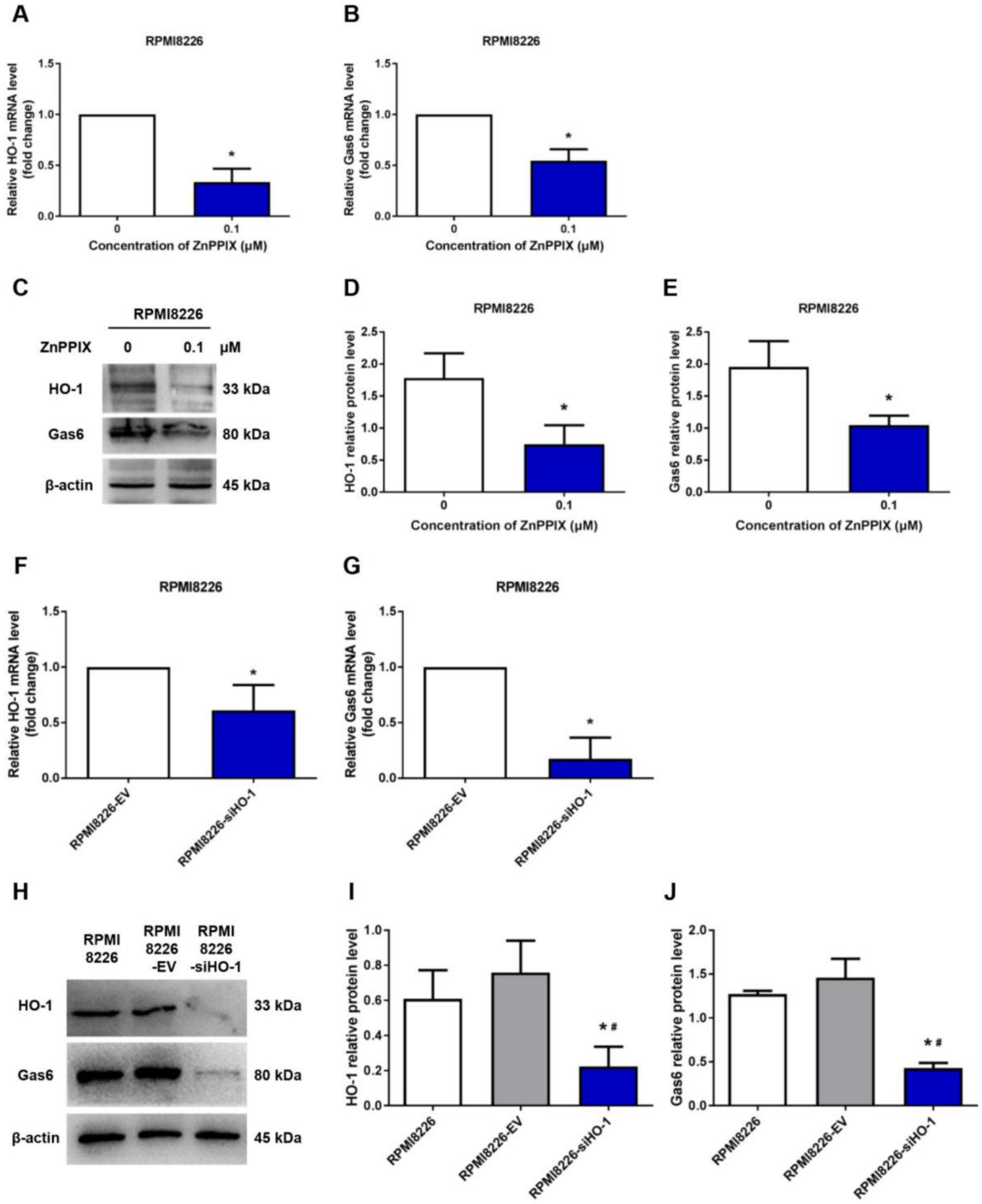

Supplementary Figure 2. HO-1 inhibition downregulates Gas6 expression in RPMI8226cells. (**A**, **B**) mRNA expression of HO-1 and Gas6 in RPMI8226cells were measured by qRT-PCR. (**C–E**) Western blot and semi-quantitative analysis of HO-1 and Gas6 protein levels in RPMI8226 cells-treated with ZnPPIX for 24 h. (**F**, **G**) HO-1 and Gas6 mRNA levels in RPMI8226 cells were measured by qRT-PCR after transfection with empty vector (EV) and HO-1 siRNA. (**H–J**) The effects of HO-1 knockdown on Gas6 protein expression level was shown in RPMI8226 cells. Data are expressed as mean ± SD (n = 4). **P*< 0.05 vs. RPMI8226; $^{\#}$*P*< 0.05 vs. RPMI8226-EV.
