# Supplementary material for: Heme oxygenase-1 inhibition mediates Gas6 to enhance bortezomib-sensitivity in multiple myeloma via ERK/STAT3 axis
Source: Aging (Albany NY). 2020 Apr 16;12(8):6611–29. doi: 10.18632/aging.102996 (PMC7202511; doi:10.18632/aging.102996)
Supplement: Supplementary Table 1 [file aging-12-102996-s001..pdf]

SUPPLEMENTARY TABLE

Supplementary Table 1. The characteristics of patients with multiple myeloma.

Patient	Age (years)	Sex	Serumβ 2M(mg/L)	Serum Albumin (g/L)	ISS stage
1	54	Female	1.98	44.4	I
2	50	Female	2.96	46.7	I
3	80	Female	2.97	42	I
4	50	Male	1.57	36.9	I
5	59	Male	3.02	37.9	I
6	68	Female	3.14	29.7	II
7	71	Female	4.07	38.7	II
8	43	Male	1.93	39.3	II
9	60	Male	6.5	32.7	II
10	88	Male	4.37	35.2	II
11	68	Male	3.7	42	II
12	47	Male	3.84	39.6	II
13	74	Female	3.8	36	II
14	47	Male	3.44	29.5	II
15	63	Male	4.95	32.4	II
16	64	Male	8.43	31.9	III
17	59	Male	7.93	30.5	III
18	51	Female	15.34	37.6	III
19	61	Male	11.67	20.7	III
20	65	Female	14.86	33.5	III
21	55	Male	6.97	39.6	III
22	66	Male	12.93	34.3	III
23	63	Male	11.34	31.8	III
24	53	Male	12.09	37.6	III
25	53	Female	11.87	40.2	III
26	67	Female	8.66	41.8	III
27	60	Male	9.74	25.5	III
28	51	Female	8.32	36.8	III
29	70	Male	9.01	35.2	III
30	61	Female	15.46	29.6	III

All patients were newly diagnosed with multiple myeloma and had not received hematology-related treatment.
